# The proportion and clinical characteristics of patients with stroke who returned to work six months after discharge from a convalescent rehabilitation ward: a descriptive study

**DOI:** 10.3389/fresc.2025.1613916

**Published:** 2025-07-22

**Authors:** Takashi Yamamoto, Yoshitaka Wada, Hirofumi Maeda, Daisuke Matsuura, Satoshi Hirano, Seiko Shibata, Masahiko Mukaino, Yohei Otaka

**Affiliations:** ^1^ Department of Rehabilitation Medicine School of Medicine, Fujita Health University, Aichi, Japan; ^2^ Department of Rehabilitation Medicine, Hokkaido University Hospital, Hokkaido, Japan

**Keywords:** activities of daily living, cerebrovascular disorders, international classification of functioning, disability and health, return to work, rehabilitation

## Abstract

**Background:**

The economic burden on individuals with stroke is a major concern, and measures to mitigate the negative effects of stroke on labor productivity are imperative. However, few studies have explored the return to work (RTW) of individuals with stroke after their discharge from rehabilitation wards. We therefore aimed to explore the proportion of patients with stroke who returned to work after discharge from a convalescent rehabilitation ward and to explore the characteristics of patients with stroke who achieve RTW compared to those who do not.

**Methods:**

This descriptive study was conducted in a convalescent rehabilitation ward at a university hospital in Japan. It included patients with stroke in the working-age population (15–64 years) who worked before the onset and were discharged from the rehabilitation ward to their homes between January 2018 and April 2022. The participants were required to respond to a questionnaire, which was sent by mail, and the RTW status at 6 months after discharge from the rehabilitation ward was investigated. They were classified into RTW and non-RTW groups, and their characteristics were compared between the groups.

**Results:**

Fifty-nine patients [mean (SD) age 53.0 (9.0) years; 42 men] among 125 who met the criteria returned the questionnaire, and their data were included in the analysis. Thirty-nine individuals [66.1%; mean (SD) age 53.0 (8.2) years; 31 men] achieved RTW. Compared to the non-RTW group, the RTW group had significantly higher total functional independence measure (FIM) scores at admission (*p* = 0.046) and discharge (*p* < 0.001), a significantly shorter duration of ward stay during hospitalization (*p* = 0.002), and a significantly smaller proportion of patients with aphasia (*p* = 0.019).

**Conclusion:**

Two-thirds of the patients in this study population had achieved RTW at 6 months after discharge from the convalescent rehabilitation ward. Patients who achieved RTW had better motor function and FIM scores at discharge than those who did not.

## 1 Introduction

Stroke is a major cause of death and disability worldwide (1). Young adults aged 20–64 years, the working-age population, constitute 31% of incident stroke cases globally (2). Significant increases have been reported in the number of prevalent cases, total deaths, and disability-adjusted life years due to stroke in this population (3). Furthermore, the economic burden on individuals with stroke is a major concern (4). Thus, implementation of measures to mitigate the negative effects of stroke on labor productivity is imperative.

Return to work (RTW) plays a key role in the rehabilitation of working-age patients with stroke. For the affected individual, not working is a significant factor for not being satisfied with life (5), and it is also a problem for governments and employers (6). The reported overall RTW rate after first stroke is 50.9%, varying from 33% to 64% in different regions, and it is 42% in Asia (7). Additionally, factors associated with RTW include hemorrhagic stroke, sex (male), occupation (white collar worker), independence in activities of daily livings (ADLs), and milder stroke severity (7). However, the heterogeneity among studies on RTW does not allow for the generalization of results.

Although the authors of many studies have reported the RTW of individuals with stroke, only a few have reported on that after discharge from rehabilitation wards (8–12). In those studies, the participants' ages, duration before RTW, and outcomes varied. Furthermore, although ADLs is one of the most common prognostic factors for RTW in patients with stroke in general, few researchers have reported an association between ADLs at discharge from rehabilitation wards and RTW (8, 9). A retrospective cohort study revealed that the modified Barthel Index did not significantly differ between the RTW and non-RTW groups (8), whereas another retrospective cohort study reported that the Barthel Index at discharge significantly differed between these two groups (9). Thus, the factors that affect RTW in patients who are discharged from rehabilitation wards remain unclear. Given that the patients with stroke who are admitted to rehabilitation wards may have relatively severe physical impairments and lower levels of ADLs, exploring the associated factors that contribute to RTW in these patients is important.

In this study, we aimed to investigate the proportion of patients who achieved RTW after 6 months from discharge from a convalescent rehabilitation ward and to explore the clinical characteristics of individuals with stroke who achieved RTW compared to those who did not.

## 2 Materials and methods

### 2.1 Study design and setting

This descriptive study was conducted in a convalescent rehabilitation ward at the Fujita Health University Hospital, Aichi, Japan. The study protocol was approved by the Ethics Committee of the Fujita Health University, and the study is reported in accordance with the Strengthening the Reporting of Observational Studies in Epidemiology guidelines (13). The requirement for informed consent was waived owing to the retrospective study design, and individuals who did not opt out were included in the study.

The convalescent rehabilitation ward was specialized for rehabilitation covered by medical insurance and was established in April 2000 in Japan. In the case of stroke, the patients can stay in the ward for up to 180 days, and they can undergo sessions for a maximum of 3 h per day, consisting of physical, occupational, and speech-language therapies, if indicated. The rehabilitation program was tailored to the specific needs of each patient, including range-of-motion, muscle-strengthening, and gait training as well as training for ADLs.

### 2.2 Participants

Patients with stroke who were hospitalized in the rehabilitation ward and discharged between January 2018 and April 2022 were enrolled. We only included individuals aged 15–64 years—that is, people in their working-age—who worked before stroke and were discharged to home. A follow-up questionnaire was routinely sent to all patients at 6 months after discharge from the rehabilitation unit. The questionnaire, which included a question about their employment status, was used for the present study.

### 2.3 Outcomes

The primary outcome was the proportion of patients who returned to work at 6 months after discharge from the convalescent rehabilitation ward. Based on the International Classification of Functioning, Disability and Health (ICF), the respondents were classified into the following six categories: (1) work for remuneration without special consideration or supportive devices; (2) work hours, workload, supportive devices, and supportive environments are necessary; (3) work is limited and requires some support from others; (4) work is limited and requires considerable support from others; (5) not able to work at all; and (6) none of the above, or not necessary. We defined 1–4 as RTW and 5–6 as non-RTW. Occupations before the onset of stroke were classified based on the International Standard Classification of Occupations, ISCO-08 (14).

The clinical characteristics assessed included age, sex, stroke type, hemiparetic side, first-ever or recurrence of stroke, aphasia, time from stroke onset to admission to the rehabilitation ward, length of ward stay, stroke impairment assessment set (SIAS) score as the comprehensive evaluation of motor impairments (15) at discharge, and functional independence measure (FIM) score at admission and discharge. The data on these clinical characteristics were collected from medical records.

The FIM is a scale for measuring ADLs that consists of 13 motor items and five cognitive items (16, 17). The motor subscore ranges from 13 to 91, whereas the cognitive subscore ranges from 5 to 35. Higher scores indicate higher levels of ADLs. The validity and reliability of this scale have been previously confirmed (18). The FIM effectiveness was calculated as follows: (FIM score at discharge – FIM score at admission)/(126 – FIM score at admission) (19). The FIM score was recorded at admission and at discharge by the therapists in charge of the patients who were well trained in scoring the FIM.

### 2.4 Statistical analysis

Baseline characteristics were compared between the non-RTW and RTW groups using the Mann–Whitney U or chi-squared test, depending on the type of variable. The total FIM score, FIM motor subscore, FIM cognitive subscore, FIM effectiveness, and duration of ward stay were compared between the non-RTW and RTW groups using the Mann–Whitney *U*-test. Pre-onset occupational classification was compared between the RTW and non-RTW groups using Fisher's exact test. Any *p*-values <0.05 were considered statistically significant. As this was an exploratory analysis, no adjustments for multiple comparisons were made. R (version 4.1.0; The R Project for Statistical Computing, Vienna, Austria) was used to perform all statistical analyses.

## 3 Results

**Figure 1:**
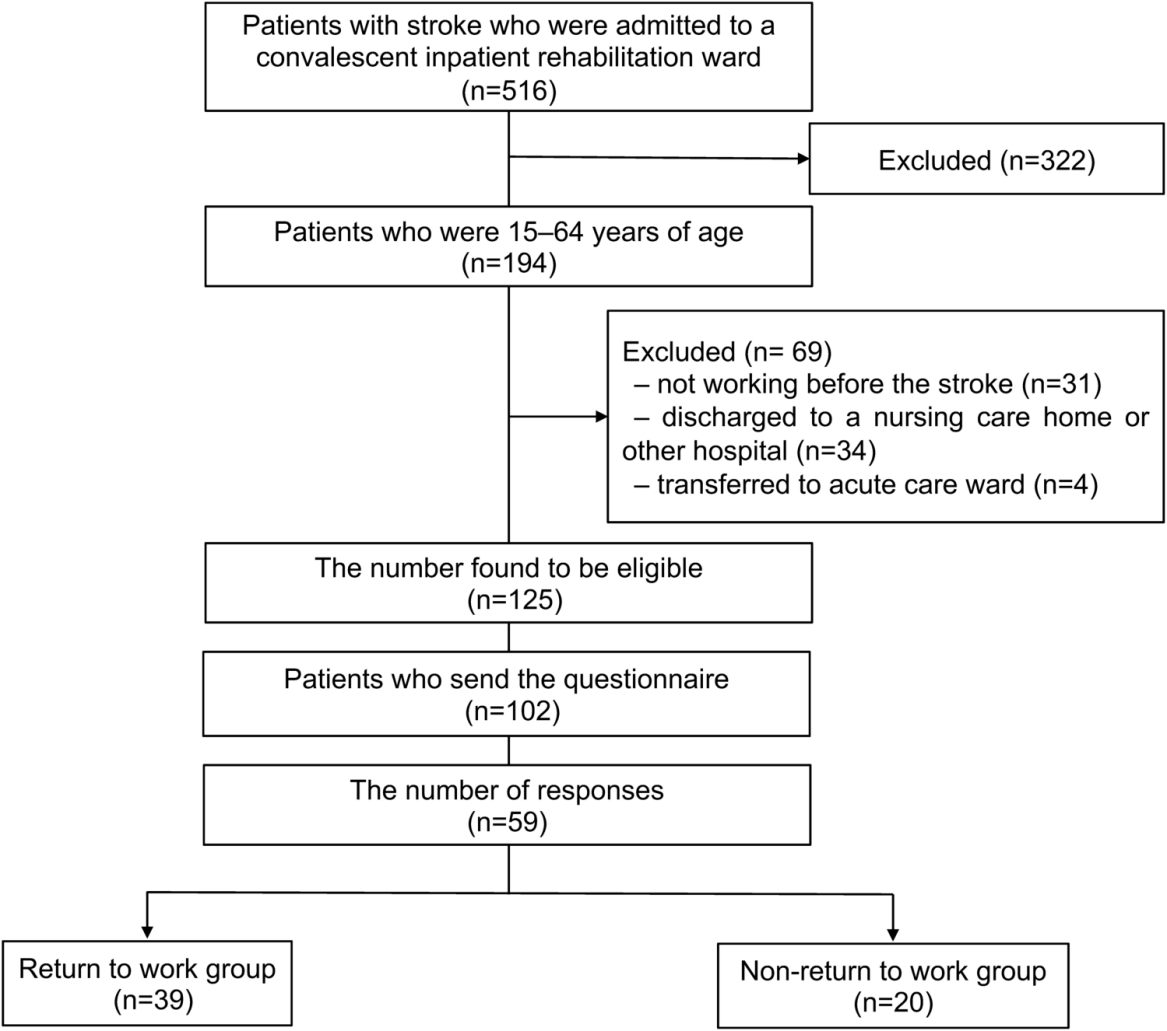
Study flow chart.

Among 516 patients with stroke who were admitted to the convalescent inpatient rehabilitation ward during the study period, 125 met the inclusion criteria. Among those, responses were obtained from 59 (47.2%) patients [mean age 53.0 (9.0) years, 42 male patients], and their data were included in the analysis. Thirty-nine out of 59 patients with stroke (66.1%) achieved RTW (Figure 1). The participant characteristics are shown in Table 1. The average age of the patients with RTW was 53.0 (8.2) years, with 79.5% were males (31/39), and 59.0% (23/39) had cerebral hemorrhage. Left hemiparesis (19/39, 48.7%) was more common than right hemiparesis (13/39), and aphasia was present in 4 patients (10.2%).

**Table 1:** Comparison of patient characteristics between those who returned to work and those who did not.

Characteristic	Total (*N* = 59)	RTW group (*n* = 39)	Non-RTW group (*n* = 20)	*p*-value
Age, years	53.0 (9.0)	53.0 (8.2)	53.0 (10.8)	0.689
Sex, male/female, *n*	42/17	31/8	11/9	0.096
Stroke type, cerebral infarction/cerebral hemorrhage/subarachnoid hemorrhage, *n*	20/29/10	10/23/6	10/6/4	0.094
Side of hemiparesis, right/left/bilateral/none, *n*	22/27/3/7	13/19/1/6	9/8/2/1	0.342
First-ever/recurrence, *n*	56/3	39/0	17/3	0.063
Aphasia, *n* (%)	12 (20.3)	4 (10.2)	8 (40.0)	0.019
Days from onset to the admission to the rehabilitation ward	28.9 (19.5)	24.6 (16.5)	37.4 (21.9)	0.017
Length of ward stay, days	28.9 (19.5)	48.7 (29.4)	80.9 (39.0)	0.002
Stroke impairment assessment scale at discharge				
Subtotal motor items score	21 (18.5–25)	21 (20–25)	19.5 (16.5–23)	0.016
Knee-mouth score	4 (4–5)	4 (4–5)	4 (3–5)	0.033
Finger function score	4 (3.5–5)	5 (4–5)	4 (2–5)	0.044
Hip flexion score	4 (4–5)	5 (4–5)	4 (3–5)	0.028
Knee extension score	4 (4–5)	5 (4–5)	4 (4–5)	0.209
Foot pat score	4 (3.5–5)	4 (4–5)	4 (2–4.3)	0.022
FIM at admission				
Motor subscore	56.9 (24.6)	60.9 (23.1)	49.1 (25.7)	0.082
Cognitive subscore	26.5 (8.4)	28.7 (6.8)	22.4 (9.7)	0.019
Total score	83.5 (31.6)	89.7 (28.1)	71.5 (34.5)	0.046
FIM at discharge				
Motor subscore	85.8 (9.7)	88.5 (5.0)	80.6 (13.7)	<0.001
Cognitive subscore	32.3 (4.8)	33.9 (2.3)	29.3 (6.7)	<0.001
Total score	118.2 (13.7)	122.4 (6.1)	109.9 (19.4)	<0.001
FIM effectiveness				
Motor subscore	0.84 (0.24)	0.87 (0.25)	0.79 (0.20)	0.010
Cognitive subscore	0.56 (0.42)	0.61 (0.42)	0.45 (0.38)	0.104
Total score	0.82 (0.24)	0.87 (0.22)	0.71 (0.22)	<0.001

Data are presented as mean (standard deviation) or median (interquartile range) unless otherwise specified. FIM, functional independence measure; RTW, return to work.

In comparisons between RTW and non-RTW groups (Table 1), age, sex, hemiparetic side, and stroke type did not significantly differ between the groups (*p* > 0.05). The non-RTW group had a longer duration from onset to admission to the rehabilitation ward [mean (SD) 37.4 (21.9) vs. 24.6 (16.5) days, *p* = 0.017] and longer length of ward stay [mean (SD) 80.9 (39.0) vs. 48.7 (29.4) days, *p* = 0.002]. The subtotal motor items of the SIAS [median (interquartile range) 21 (20–25) vs. 19.5 (16.5–23), *p* = 0.016] and all motor items at discharge were significantly better in the RTW group than in the non-RTW group (all *p*-values <0.05), except for the knee-extension item in the SIAS (*p* = 0.209).

The total FIM scores were better in the RTW group than in the non-RTW group on admission [mean (SD) 89.7 (28.1) vs. 71.5 (34.5), *p* = 0.046] and at discharge [mean (SD) 122.4 (6.1) vs. 109.9 (19.4), *p* < 0.001], and other subscores were also better in the RTW group (all *p*-values <0.05) except for the motor subscore at admission (*p* = 0.082). The FIM effectiveness was better in the RTW group than in the non-RTW group [mean (SD) 0.87 (0.22) vs. 0.71 (0.22), *p* < 0.001], although the FIM effectiveness for the cognitive items was not statistically significant (*p* = 0.104).

In addition, the proportion of patients with aphasia was significantly higher in the non-RTW group than in the RTW group (40% vs. 10.2%, *p* = 0.019). The job classifications of the participants before stroke onset did not significantly differ between the groups (*p* = 0.285; Table 2).

**Table 2:** Job classification before stroke onset of participants who returned to work and those who did not.

Major groups	Total (*N* = 59)	RTW group (*n* = 39)	Non-RTW group (*n* = 20)
Managers	12	9 (23.1)	3 (15.0)
Professionals	14	8 (20.5)	6 (30.0)
Technicians and associate professionals	2	2 (5.1)	0 (0.0)
Clerical support workers	7	6 (15.4)	1 (5.0)
Services and sales workers	9	5 (12.8)	4 (20.0)
Skilled agricultural, forestry, and fishery workers	1	0 (0.0)	1 (5.0)
Craft and related trade workers	6	2 (5.1)	4 (20.0)
Plant and machine operators and assemblers	5	5 (12.8)	0 (0.0)
Elementary occupations	3	2 (5.1)	1 (5.0)
Armed forces	0	0 (0.0)	0 (0.0)

Values are indicated as number (percentage). RTW, return to work.

## 4 Discussion

The present study revealed that 66.1% of the included patients achieved RTW at 6 months after discharge from the convalescent rehabilitation ward. Those who achieved RTW had a shorter duration between stroke onset and admission to the rehabilitation ward, shorter length of stay in the ward, higher FIM score at admission and discharge, and higher FIM effectiveness. They also had milder paralysis at discharge. Furthermore, the proportion of patients with aphasia was lower in the RTW group than in the non-RTW group.

The proportion of patients who achieved RTW in this study was consistent with that found in previous studies (8, 11, 12). In this study, 66 patients did not respond to the questionnaire. We calculated the minimum and maximum RTW rates assuming that all nonresponding patients did not RTW and all patients did RTW, respectively; this yielded estimated RTW rates ranging from 38.2% (39/102) to 80.3% (82/102). Based on these assumptions, the range of percentages of patients who achieved RTW considerably overlapped with—despite being somewhat higher than—those found in previous studies (8, 11, 12), in which the percentage of patients who achieved RTW at 6 months after discharge from the rehabilitation ward ranged from 18% to 53.6%. Retrospective studies with longer follow-up periods revealed rates of 7% of patients RTW at 1 year (10) and 32.1% RTW at 3 years after discharge from rehabilitation wards (9). These differences in proportions could be due to heterogeneity in methodologies. For example, in terms of age, the inclusion criteria used in previous studies were 15–64 years (11), 21–65 years (8), 18–65 years (9), and <65 years (10). In one study, the researchers did not restrict the age of the participants (12). All studies included all patients discharged from rehabilitation wards, although our study included only those who were discharged and returned to their homes. Therefore, the results may differ depending on the patient-selection method used.

Theoretically, patients with less severe strokes and who have recovered sufficiently physically and cognitively are more likely to return to work, which is also found in many previous reports (7, 9, 11). Consistent with the theoretical thinking and previous reports, this study found that those who achieved RTW had higher ADLs at admission and discharge and higher motor function at discharge than those who did not. In addition, the RTW group showed a shorter length of stay in the rehabilitation ward and greater improvement in ADL than the non-RTW group. These findings indicate that patients with better motor function and ADLs and better progress are more likely to achieve RTW. However, the SIAS motor item scores and FIM score were sufficiently high even in the non-RTW group in the present study. This implies that most of these individuals did not achieve RTW even though they had achieved sufficient function and independence in ADLs. An important finding here is that the FIM cognitive subscores at admission and discharge were significantly higher in the RTW group than in the non-RTW group. In previous studies (8, 9), the researchers have used the Barthel Index and its modifications, which did not include cognitive status; therefore, details on the relationship between cognitive status and RTW were not provided. Notably, the proportion of patients with aphasia was higher in the non-RTW group than in the RTW group in our study, which is consistent with the results of previous studies (7, 20). Our findings therefore indicate that, even with relatively high levels of motor function and ADLs at discharge, lower cognitive function, including language ability, is associated with lower levels of RTW achievement.

In addition to the patients' ability, the socioeconomical background would also have an impact on RTW. Especially, job content has generally been identified as an important factor for RTW in patients with stroke (7, 21). In contrast to the previous reports, the present study found no significant differences in occupational classification prior to stroke onset between RTW and non-RTW groups. Further research is needed to understand how job content affects RTW after a stroke.

The clinical implications of this study suggest that clinicians should focus on effective interventions for RTW in individuals with stroke who are considering working. Several variables that act as barriers to RTW are modifiable. Although high-quality trials are still lacking to substantiate recommendations for specific vocational rehabilitation programs to increase RTW rates after stroke (22), initiating such a program during hospitalization may be a practical approach. A model of vocational rehabilitation for RTW during hospitalization should be established in Japan, as different countries have different forms of support in such an approach. The second approach is to adjust the work environment. A flexible work environment and supportive social networks were cited as factors that encouraged a return to paid employment (23). Adjustments to the work environment, including changes in the work content, can be beneficial for individuals with disabilities.

This study has a few limitations. First, it was a single-center, retrospective study conducted in Japan. Therefore, the generalizability of the results to other institutions and countries may be limited. Second, selection bias could have been introduced, as only those patients who were discharged home and could complete the questionnaire were included. Patients with lower functional statuses may have been excluded, resulting in a higher estimate of the RTW ratio. Further, the non-RTW participants could have been possibly assessed as having better overall functional characteristics. Third, information on non-medical factors that may affect the likelihood of RTW, such as family preferences and supports for RTW and cooperation with occupational physicians, is lacking. Fourth, the limited sample size precluded multivariable modeling. Therefore, the findings should be interpreted with caution because we did not statistically adjust for potential confounding variables. Future studies should examine the quality of life, satisfaction, employment status, types of work, and labor income of patients with stroke who have achieved RTW.

In conclusion, the proportion of patients who achieved RTW at 6 months after discharge from the convalescent rehabilitation ward was 66.1%. Patients who achieved RTW had a better functional status at discharge than those who did not. This study offers a valuable benchmark in the context of limited available evidence. A more detailed national survey is required to explore the realities and factors behind RTW and to develop effective measures to promote RTW.

## Data Availability

The datasets presented in this article are not readily available because due to the nature of this research, participants of this study did not agree for their data to be shared publicly, so supporting data is not available. Requests to access the datasets should be directed to Yoshitaka Wada, yoshi1201.wada@gmail.com.
